# Structural studies of P-type ATPase–ligand complexes using an X-ray free-electron laser

**DOI:** 10.1107/S2052252515008969

**Published:** 2015-06-11

**Authors:** Maike Bublitz, Karol Nass, Nikolaj D. Drachmann, Anders J. Markvardsen, Matthias J. Gutmann, Thomas R. M. Barends, Daniel Mattle, Robert L. Shoeman, R. Bruce Doak, Sébastien Boutet, Marc Messerschmidt, Marvin M. Seibert, Garth J. Williams, Lutz Foucar, Linda Reinhard, Oleg Sitsel, Jonas L. Gregersen, Johannes D. Clausen, Thomas Boesen, Kamil Gotfryd, Kai-Tuo Wang, Claus Olesen, Jesper V. Møller, Poul Nissen, Ilme Schlichting

**Affiliations:** a Department of Molecular Biology and Genetics, Centre for Membrane Pumps in Cells and Disease – PUMPkin, Danish National Research Foundation, Aarhus University, Gustav Wieds Vej 10c, 8000 Aarhus C, Denmark; b Max Planck Institute for Medical Research, Jahnstrasse 29, 69120 Heidelberg, Germany; c Rutherford Appleton Laboratory, ISIS Facility, Chilton, Didcot OX11 0QX, England; d Department of Physics, Arizona State University, Tempe, AZ 85287, USA; e Linac Coherent Light Source, LCLS, SLAC National Accelerator Laboratory, 2575 Sand Hill Road, Menlo Park, CA 94025, USA; f Department of Biomedicine, Aarhus University, Ole Worms Allé 3, 8000 Aarhus C, Denmark; g Department of Neuroscience and Pharmacology, University of Copenhagen, Blegdamsvej 3B, 2200 Copenhagen, Denmark; h DANDRITE, Nordic-EMBL Partnership for Molecular Medicine, Aarhus University, Gustav Wieds Vej 10C, DK-8000 Aarhus C, Denmark

**Keywords:** XFEL, P-type ATPases, ligand screening, serial femtosecond crystallography

## Abstract

Membrane proteins are key players in biological systems, mediating signalling events and the specific transport of *e.g.* ions and metabolites. Consequently, membrane proteins are targeted by a large number of currently approved drugs. Understanding their functions and molecular mechanisms is greatly dependent on structural information, not least on complexes with functionally or medically important ligands. Structure determination, however, is hampered by the difficulty of obtaining well diffracting, macroscopic crystals. Here, the feasibility of X-ray free-electron-laser-based serial femtosecond crystallography (SFX) for the structure determination of membrane protein–ligand complexes using microcrystals of various native-source and recombinant P-type ATPase complexes is demonstrated. The data reveal the binding sites of a variety of ligands, including lipids and inhibitors such as the hallmark P-type ATPase inhibitor orthovanadate. By analyzing the resolution dependence of ligand densities and overall model qualities, SFX data quality metrics as well as suitable refinement procedures are discussed. Even at relatively low resolution and multiplicity, the identification of ligands can be demonstrated. This makes SFX a useful tool for ligand screening and thus for unravelling the molecular mechanisms of biologically active proteins.

## Abstract 2

The structure determination of P-type ATPase–ligand complexes from microcrystals by serial femtosecond crystallography using a free-electron laser is described. The feasibility of the method for ligand screening is demonstrated, and SFX data quality metrics as well as suitable refinement procedures are discussed.

## 1. Introduction   

Membrane protein crystallization and structure determination are subject to multiple challenges, such as low yields, protein instability and small crystals that are difficult to cryocool. The production of well diffracting macroscopic crystals can be a major bottleneck, and the optimization of crystals often requires the time-consuming and expensive production of large amounts of pure and stable protein samples. After the successful determination of a first structure of a given target, efforts often multiply in the quest to obtain further structures with different bound compounds, such as stabilizers of different conformations and/or compounds used for structure-based drug development.

The emerging method of serial femtosecond crystallo­graphy (SFX) at X-ray free-electron laser (XFEL) sources enables high-resolution diffraction data collection from nanocrystal or microcrystal suspensions injected into the XFEL beam in their mother liquor at room temperature. This circumvents the need for the time- and sample-demanding optimization of large single crystals and their tedious handling through cryoprotection, mounting and cryocooling required for synchrotron-based approaches. We tested the feasibility of SFX for the structure determination of ligand complexes of membrane proteins using microcrystals of P-type ATPases derived from both natural and recombinant sources bound to different ligands.

P-type ATPases are ubiquitous, energy-dependent membrane transporters that are present in all kingdoms of life and constitute important drug targets both in humans and in microbial pathogens owing to their vital role in the establishment and maintenance of transmembrane ion gradients (Yatime *et al.*, 2009 ▸; Bublitz *et al.*, 2011 ▸). The study of the interactions of ATPase with regulating ligands or inhibitors is not only instrumental for the general understanding of enzyme function (for example, vanadate or fluoride compounds mimicking phosphoryl transition states, non­hydrolyzable nucleotide analogues, lipids or stabilizers of known catalytic conformations), but also for efforts in structure-based drug development targeting P-type ATPase activities in, for example, pathogens or cancer cells. For example, tissue-specific inhibition of the sarco(endo)plasmic reticulum Ca^2+^-ATPase SERCA is currently being clinically tested as a strategy against hepatoma and prostate cancer by induced apoptosis (Denmeade *et al.*, 2003 ▸). In addition to human targets such as SERCA, the Na^+^,K^+^-ATPase, the gastric H^+^,K^+^-ATPase or heavy metal-transporting ATPases, fungal plasma membrane H^+^-ATPases as well as P-type ATPases from the malaria pathogen *Plasmodium falciparum* are examples of targets for the development of new drugs (Rottmann *et al.*, 2010 ▸; Arnou *et al.*, 2011 ▸). Here, we demonstrate the potential of SFX for ligand characterization by analyzing microcrystals of three different P-type ATPase–ligand complexes: SERCA1a from rabbit leg muscle bound to (i) the nonhydrolyzable ATP analogue 5′-adenylyl(β,γ-methylene)diphosphonate (AMPPCP) and calcium (SERCA–Ca_2_–AMPPCP; (ii) to the fluorescent ATP analogue 2′,3′-*O*-(2,4,6-tri­nitrophenyl)adenosine 5′-triphosphate (TNPATP) and orthovanadate (SERCA–VO_3_–TNPATP); and (iii) as a model protein for heavy metal-transporting ATPases, *Shigella sonnei* Zn^2+^-ATPase ZntA (SsZntA) produced recombinantly in *Escherichia coli* and bound to aluminium tetrafluoride (SsZntA–AlF_4_).

SFX exploits the high intensity and femtosecond duration of XFEL pulses for the acquisition of radiation damage-free diffraction data in the so-called ‘diffraction before destruction’ approach (Chapman *et al.*, 2011 ▸, 2014 ▸; Boutet *et al.*, 2012 ▸). A consequence of this approach is, however, that only a single still image can be collected from each crystal, requiring a steady supply of fresh crystals. This can be provided by means of microjets (Weierstall *et al.*, 2012 ▸, 2014 ▸; Sierra *et al.*, 2012 ▸), which allow the delivery of crystals to the XFEL interaction region at room temperature and in their crystallization medium, including lipidic mesophases such as sponge or cubic phase media. Thus, diffraction data are collected in a serial fashion, with each diffraction image derived from a different, randomly oriented microcrystal. Owing to variations in the size, diffraction properties, unit-cell parameters and mosaicity of the single crystals, as well as fluctuations in the X-ray beam parameters (pulse energy and spectral distribution) and variations in how well the crystals intersect the X-ray focus at the time of exposure, a large number of integrated diffraction patterns are usually necessary to provide a sufficiently high multiplicity of observations for each reflection to provide a reliable conversion of the partial intensities to accurate structure-factor amplitudes in a scheme called Monte Carlo integration (Kirian *et al.*, 2010 ▸, 2011 ▸). However, given the difficulties in obtaining large quantities of membrane-protein samples, this is not always possible (Kern *et al.*, 2012 ▸, 2013 ▸; Johansson *et al.*, 2013 ▸), as was also the case for the data presented here. We show that even at low resolution and with low multiplicity, SFX data can provide valuable structural information on a variety of ligands bound to a known structure, and we discuss general considerations for SFX data collection and analysis.

## 2. Methods   

### 2.1. Preparation and microcrystallization of P-type ATPases   

All reagents and the samples of either purified protein or membrane preparations were shipped to the LCLS and the microcrystals were grown on site.

#### 2.1.1. Ca^2+^-ATPase   

115 mg Ca^2+^-ATPase protein in rabbit sarco(endo)plasmic reticulum vesicles, prepared according to established procedures (Andersen *et al.*, 1985 ▸), was used as the starting material. For the Ca_2_–AMPPCP form, the ATPase was solubilized in a buffer consisting of 94.6 m*M* MOPS/KOH pH 6.8, 80 m*M* KCl, 20% glycerol, 2.5 m*M* MgCl_2_, 0.88 m*M* AMPPCP, 8.87 m*M* CaCl_2_, 22.3 mg ml^−1^ octaethylene glycol monododecyl ether (C_12_E_8_). The solubilized protein at a concentration of 10 mg ml^−1^ was mixed in equal amounts with crystallization buffer (21.5% polyethylene glycol 6000, 200 m*M* sodium acetate, 15% glycerol, 4% *tert*-butanol, 5 m*M* β-mercaptoethanol) in batches of 50–100 µl in PCR reaction tubes. Microcrystals with approximate dimensions of 10 × 5 × 5 µm grew within 2–3 d at 19°C.

For crystallization of the SERCA–VO_3_–TNPATP complex, the protein was prepared as above, apart from pre-incubation for 1.5 h with sodium orthovanadate before the addition of TNPATP and detergent solubilization. The final buffer composition was 100 m*M* MOPS/KOH pH 6.8, 80 m*M* KCl, 20% glycerol, 0.25 m*M* MgCl_2_, 1.5 m*M* EGTA, 1 m*M* VO_4_^3−^, 0.4 m*M* TNPATP, 22.3 mg ml^−1^ C_12_E_8_. Batches of 20 µl containing equal amounts of protein solution (10 mg ml^−1^) and crystallization buffer (28% polyethylene glycol 2000 monomethyl ether, 10% glycerol, 100 m*M* NaCl, 3% *tert*-butanol) were mixed and rod-shaped microcrystals of approximately 2 × 2 × 10–50 µm in size grew within 3 d at 19°C. The batches, yielding a total volume of 8 ml for SERCA–Ca_2_–AMPPCP and 4.27 ml for SERCA–VO_3_–TNPATP, were combined, yielding microcrystalline sediments constituting ∼30–40% of the batch volume for SERCA–Ca_2_–AMPPCP and ∼80% of the batch volume for SERCA–VO_3_–TNPATP. The combined batch was vortexed for 3.5 min in the case of SERCA–VO_3_–TNPATP to break the long needle-shaped crystals into shorter pieces, and all samples were filtered (see below) immediately before data collection.

#### 2.1.2. Zn^2+^-ATPase   

ZntA from *S. sonnei* (SsZntA) was expressed in *E. coli* C41 cells induced with 1 m*M* IPTG at 20°C. The cells were resuspended on ice in 50 m*M* Tris–HCl pH 7.6, 200 m*M* KCl, 20% glycerol, 5 m*M* β-mercaptoethanol (BME), 2 µg ml^−1^ DNase I and one protease-inhibitor tablet (Sigma) per 6 l of culture and were lysed with a high-pressure homogenizer at 138 MPa. Cell debris was removed by centrifugation at 20 000*g* and 4°C for 45 min, and membranes were isolated by ultracentrifugation at 250 000*g* and 4°C for 5 h and resuspended on ice in 15 ml 20 m*M* Tris–HCl pH 7.6, 200 m*M* KCl, 20% glycerol, 5 m*M* BME, 1 m*M* MgCl_2_ per gram of membrane pellet. The membrane proteins were solubilized for 1 h at 4°C in 1%(*w*/*v*) C_12_E_8_ and unsolubilized material was removed by ultracentrifugation at 250 000*g* and 4°C. Solid KCl and imidazole were added to final concentrations of 500 and 50 m*M*, respectively, and the solution was applied onto a pre-packed 5 ml Ni^2+^–NTA column followed by a Superose 6 10/300 GL gel-filtration column using buffer *D* (20 m*M* MOPS–KOH pH 6.8, 80 m*M* KCl, 20% glycerol, 5 m*M* BME, 1 m*M* MgCl_2_, 0.15 mg ml^−1^ C_12_E_8_). The purified protein was concentrated to 8 mg ml^−1^ using a Sartorius Vivaspin 20 centrifugal concentrator (with molecular-weight cutoff 50 kDa) and frozen until further use.

SsZntA was crystallized in batch mode in the presence of 2 m*M* AlF_4_^−^, 2 m*M* EGTA and 10 µ*M* *N*,*N*,*N*′,*N*′-tetrakis-2-pyridylmethyl-ethylenediamine. Prior to crystallization, 72× CMC of C_12_E_8_ was added to the protein, and batches of 50 µl protein with 50 µl precipitant solution (100 m*M* MOPS pH 6.8, 0.5 *M* lithium acetate, 15% PEG 2K MME, 10% glycerol, 3% *tert*-butanol, 5% d-sorbitol, 5 m*M* BME) were incubated at 19°C. Rod-shaped crystals of 5 µm in length were fully grown within 2 d after setup, but in clusters surrounded by amorphous precipitate. Therefore, before injection, the microcrystal suspension of total volume 1.3 ml was repeatedly passed through a 19 mm long G.27 syringe needle with an inner diameter of 0.210 ± 0.019 mm (BD Biosciences) to homogenize the crystal suspension.

### 2.2. Injection of microcrystals into the pulsed XFEL beam   

Microcrystal suspensions were kept at 18°C and filtered through a 20 µm inline stainless-steel filter. The microcrystals were injected with an HPLC system (Shimadzu Biotech, Duisburg, Germany) into the vacuum chamber in an ∼5 µm wide liquid jet produced with a gas dynamic virtual nozzle (GDVN; DePonte *et al.*, 2008 ▸) housed in a liquid injector (Weierstall *et al.*, 2012 ▸) as described previously (Chapman *et al.*, 2011 ▸; Boutet *et al.*, 2012 ▸). In general, the sample flow rate was 12–30 µl min^−1^. A protocol for alternating sample and water flow through the jet capillary proved to be useful to prevent nozzle clogging, thus maintaining a constant hit rate and diffraction quality.

### 2.3. Collection of diffraction data   

An average of 3.75 mJ was delivered in each 50 fs pulse of 6.0 keV X-rays. The beamline transmission was 40%. Single-pulse diffraction patterns were recorded at 120 Hz using a Cornell–SLAC pixel-array detector (CSPAD; Hart *et al.*, 2012 ▸) positioned in the microfocus chamber of the CXI instrument at a distance of 130 mm from the interaction region.

### 2.4. Data processing, phasing and refinement   

Raw data streams from the CXI instrument were pre-analysed using the *CASS* software suite (Foucar *et al.*, 2012 ▸). After offset-correcting the individual images and masking bad pixels based upon the noise of the individual pixels, PostProcessor with ID 208 was used to identify Bragg spots (Foucar *et al.*, to be submitted). A selected background size of 4 × 4, which resulted in a box size of 9 × 9 pixels, was used with a local signal-to-noise ratio of 4. The minimum number of pixels contributing to a Bragg spot was set to three and the threshold to identify the highest pixel value within the box was set to 400 ADU. Identified crystal hits were saved in HDF5 format for indexing and Monte Carlo merging using *CrystFEL* (White *et al.*, 2012 ▸). Merged structure factors were exported in CCP4 format for structural analysis.

Phases for SERCA–Ca_2_–AMPPCP and SERCA–VO_3_–TNPATP were obtained by molecular replacement (MR) with *Phaser* (McCoy *et al.*, 2007 ▸) using the *PHENIX AutoMR* implementation (Adams *et al.*, 2010 ▸). The search models were PDB entry 3n8g for SERCA–Ca_2_–AMPPCP and PDB entry 3n5k for SERCA–VO_3_–TNPATP (Bublitz *et al.*, 2013 ▸), with all ligands removed prior to MR procedures. In order to diminish the model bias from the Ca^2+^ and AMPPCP ligands which had been included in the refinement of the isomorphous 3n5k search model, we carried out a simulated-annealing (500 K) refinement against the original data and reset all *B* factors to the average value before using the model in the molecular-replacement procedure. All refinements were carried out using *PHENIX* (Adams *et al.*, 2010 ▸). SERCA–Ca_2_–AMPPCP was first refined by rigid-body refinement, with 13 rigid groups comprising the cytosolic A, P and N domains as well as one separate group for each of the ten transmembrane helices. The output model from the 13-group rigid-body refinement was then used as an input model for refinement of coordinates and *B* factors for all 7671 (SERCA alone, without ligands) or 7706 (including nucleotide and bound ions) atoms. To avoid overfitting, we included an optimization of the restraint weights for both geometry and *B* factors during refinement, and we used the original molecular-replacement model 3n8g as a reference for the generation of dihedral restraints, except for the final refinement round. Map files were generated with *FFT* (Read & Schierbeek, 1988 ▸) or *PHENIX* (Adams *et al.*, 2010 ▸), and figures were prepared with *PyMOL* (Schrödinger). No ligands were included in either the molecular-replacement or the initial refinement runs in order to obtain bias-free *mF*_o_ − *DF*_c_ difference electron-density maps. Where applicable, the *R*_free_ flags were copied from the data set against which the search model had originally been refined to ensure an independent cross-validation set. The SERCA–VO_3_–TNPATP structure was refined by rigid-body strategies only, using the same 13 rigid groups as above.

For the generation of a data set with scrambled reflection intensities, reflections (and their sigma values) were sorted by resolution, put into groups of ten and then randomly scrambled, but keeping the *R*_free_ flags associated with the original index *hkl*. To this end, the data were sorted by resolution using *SFTOOLS* (Hazes, unpublished work) and grouped into bins of ten reflections. The reflections were then scrambled randomly within a bin. In this way, the dependence of the intensity statistics on the resolution was preserved as much as possible while still scrambling the intensities themselves. The original reflections at a resolution higher than 5.8, 4.6, 4.0, 3.6, 3.4, 3.2, 3.0 or 2.9 Å were then replaced with the scrambled data (*CAD*, *MTZUTILS*; Winn *et al.*, 2011 ▸), yielding a set of ‘chimeric’ data sets, all extending to 2.8 Å resolution but only containing real data up to the given cutoff.

## 3. Results   

### 3.1. Microcrystallization   

Since the growth of macroscopic crystals has been the most common approach to obtain high-resolution X-ray diffraction data in the past decades, procedures aimed at maximizing crystal size have been well described (Bergfors, 2009 ▸; Chayen & Saridakis, 2008 ▸; Doublié, 2007 ▸). This is, however, not the case for protocols aiming to grow large amounts of uniformly sized microcrystals. The following section describes in detail how we established a robust and reproducible batch microcrystallization procedure for our P-type ATPase samples. Liquid microjet injection requires relatively large sample volumes, which is why we used batch approaches for crystallization. In order to establish a reproducible production of batches with uniformly sized microcrystals, we started from the vapour-diffusion hanging-drop crystallization conditions previously optimized for macroscopic crystals. In the case of SERCA–Ca_2_–AMPPCP, these macrocrystals had been used for the original structure determination (Sørensen *et al.*, 2004 ▸), whereas macrocrystals of SERCA–VO_3_–TNPATP and orthovanadate and of SsZntA–AlF_4_ had, at the time of our SFX experiments, both yielded diffraction to about 4 Å resolution at a synchrotron and no structures had been published. We systematically explored the variation of all components of the precipitant solutions (PEG, salt, glycerol, reducing agent and alcohol type and concentrations), as well as varying the ligand concentrations, batch sizes and batch-mixing procedures (manual ‘flipping’ *versus* short vortexing). We also tested the addition of immersion oil either directly on top of the batch mixture or inside the lid of the reaction tube, since hanging-drop crystallization of most P-type ATPases in our hands strongly depended on the use of immersion oil to seal the wells. Furthermore, we tested the crystallizability of SERCA preparations from rabbit sarcoplasmic reticulum, which had, for the purpose of a larger protein yield at the cost of slightly lower purity, not been purified by deoxycholate (DOC) extraction, as is usually performed when following established procedures (Møller *et al.*, 2002 ▸). In all cases, the final conditions that yielded the highest density of the most uniformly sized microcrystals in 30–100 µl batches were astonishingly similar to the starting conditions for growing macrocrystals in hanging drops: essentially, only the concentration of the precipitating agent needed to be increased (by approximately 30 and 170% for SERCA–VO_3_–TNPATP and SERCA–Ca_2_–AMPPCP, respectively), and for SsZntA–AlF_4_ the alcohol additive was changed from MPD to *t*-BuOH (see Supplementary Table S1). Varying other parameters such as salt or glycerol concentration as well as the presence or absence of immersion oil or omitting the DOC extraction step had surprisingly little effect on crystal size. The only notable factors besides the precipitant concentration were the mixing procedure (thorough manual ‘flipping’ of the tubes was superior to superficial mixing or vortexing, the former of which led to a non-uniform size distribution of crystals and the latter of which introduced foam in the detergent-containing conditions), and there was an inverse correlation of optimal glycerol and PEG concentrations: higher PEG concentrations yielded more uniform batches in combination with lower glycerol, also owing to the avoidance of the phase separation which occurred at very high PEG and glycerol concentrations.

For the measurements at the LCLS, frozen microsomes or protein samples were shipped to SLAC National Accelerator Laboratory, where the proteins were crystallized on-site before data collection. Single batches were set up in volumes of 30–100 µl, grew within 1–2 d (which is consistently faster than growing macrocrystals, which typically need 1–2 weeks to reach final size) and manually combined immediately before data collection. SsZntA–AlF_4_ microcrystals grew as clusters embedded in amorphous precipitate, which were homogenized by passing the sample repeatedly through a 400 um syringe needle before data collection. Microcrystals of SERCA–VO_3_–TNPATP grew as long needles of varying lengths and diameters, which were mechanically homogenized by vortexing before data collection with no apparent loss of diffraction quality.

### 3.2. Data collection   

SFX data were collected in June 2012 (proposal L597) using the microfocus chamber of the CXI instrument at the LCLS as described previously (Boutet *et al.*, 2012 ▸). The microcrystalline suspensions were kept at 18°C during data collection using a temperature-controlled rotating syringe pump that also prevented crystal settling (Lomb *et al.*, 2012 ▸). The microcrystals were injected into the XFEL beam using a liquid microjet injector (Weierstall *et al.*, 2012 ▸). The high concentrations of high-molecular-weight polyethylene glycols and glycerol in all samples led to occasional clogging of the injection nozzle. This problem was overcome by alternating water and sample injection at 5–10 min intervals. SFX diffraction data were collected at 6 keV photon energy (2.07 Å wavelength) at the full 120 Hz repetition rate of the LCLS using a CSPAD detector (Hart *et al.*, 2012 ▸). All diffraction images were saved, irrespective of whether or not they contained diffraction peaks. Crystal hits were identified by *CASS* (Foucar *et al.*, 2012 ▸) as images containing more than ten Bragg reflections. Hits were indexed, integrated and merged with the *CrystFEL* software suite (White *et al.*, 2012 ▸). The detector geometry was calibrated using a lysozyme SFX data set collected immediately prior to P-type ATPase data collection (data not shown). We collected three SFX data sets from P-type ATPase microcrystals: rabbit SERCA with bound calcium ions and AMPPCP (SERCA–Ca_2_–AMPPCP), SERCA with bound orthovanadate and TNPATP (SERCA–VO_3_–TNPATP) and *S. sonnei* Zn^2+^-ATPase SsZntA bound to the inhibitor aluminium tetrafluoride (SsZntA–AlF_4_).

### 3.3. SERCA–Ca_2_–AMPPCP: refinement   

**Figure 1:**
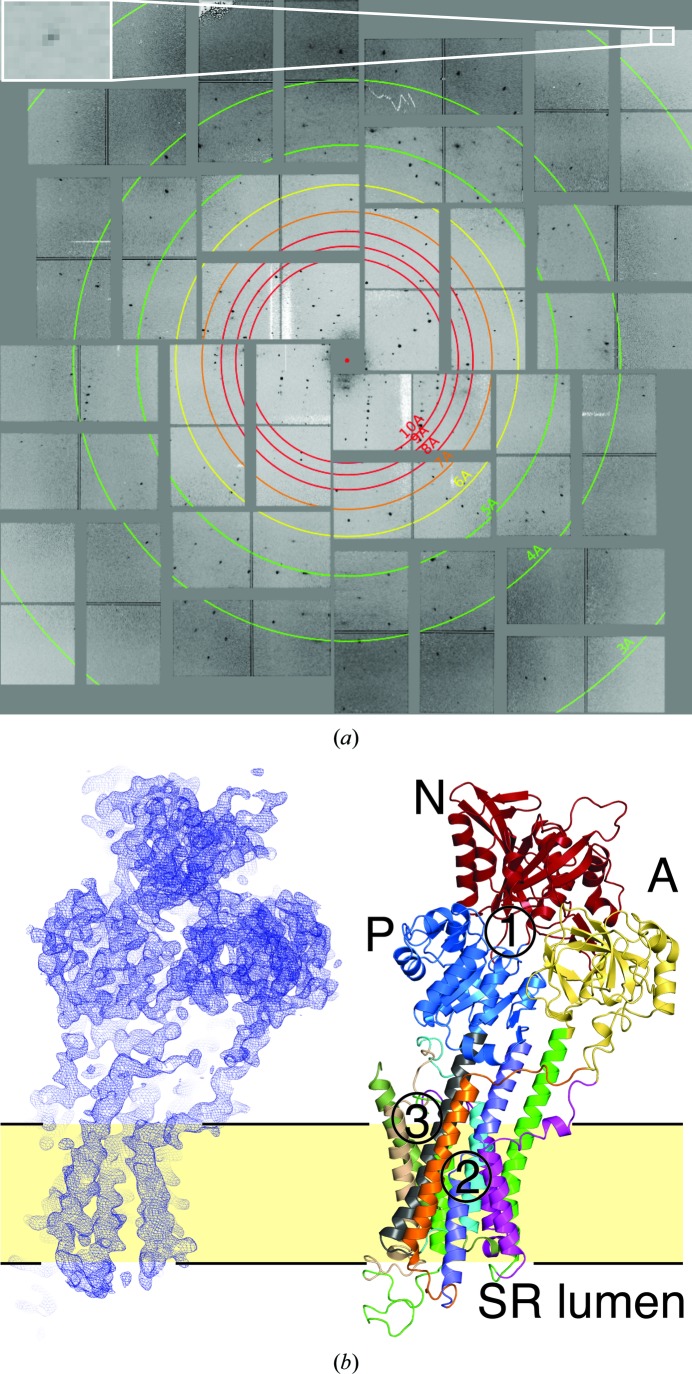
Diffraction pattern, electron density and structure of SERCA–Ca_2_–AMPPCP. (*a*) Representative diffraction pattern of SERCA–Ca_2_–AMPPCP microcrystals. The boxed inset shows a strong Bragg spot at 2.8 Å resolution {Miller index [31, −15, 24], 〈*I*/σ(*I*)〉 = 8.4}. (*b*) Left, overall 2*mF*_o_ − *DF*_c_ electron-density map (contoured at 1.0σ) after final refinement including coordinates and *B* factors for all atoms and data to 2.8 Å resolution; right, molecular model, including ten transmembrane helices and cytoplasmic N (nucleotide binding), P (phosphorylation) and A (actuator) domains. The 13 structural segments initially refined as rigid groups are shown in different colours. Ligand-binding regions are indicated by numbers: 1, AMPPCP; 2, Ca^2+^ ions; 3, lipid.

SFX diffraction images of microcrystals of SERCA–Ca_2_–AMPPCP showed diffraction beyond 3 Å resolution (Fig. 1 ▸ *a*). This is not on a par with the 2.5 Å resolution diffraction limit of the corresponding cryocooled macrocrystals at a synchrotron (Sørensen *et al.*, 2004 ▸), and may be owing to a narrow time window for optimal microcrystal quality, in which an increase in the age of the crystal sample of only 24 h already led to weaker diffraction and a higher number of overlapping diffraction patterns, indicating crystal clustering. We recorded ∼760 000 images, of which ∼23 000 (3%) were identified as hits containing a diffraction pattern; of these, 4069 (∼18%) could be indexed in *C*2, the same space group as macroscopic SERCA–Ca_2_–AMPPCP crystals. The merged data have an overall ∼17-fold multiplicity of observation. This is extremely low for SFX data analysed by *CrystFEL*, which uses Monte Carlo methods to obtain scaled and merged intensities from the still images, averaging out all fluctuations in the data-acquisition process. One has to bear in mind, however, that in SFX multiplicity refers to the number of partial intensity measurements and not of fully integrated intensities. In line with this low multiplicity, the signal-to-noise ratio drops to 1.47 at 4 Å resolution (Table 1 ▸ and Supplementary Table S2). However, it must also be considered that the signal-to-noise ratio as defined by *CrystFEL* is based on the standard deviations of the sets of individual observations of a reflection themselves, not on the signal-to-noise ratios of merged, scaled and averaged diffraction peaks representing fully recorded reflections.

**Figure 2:**
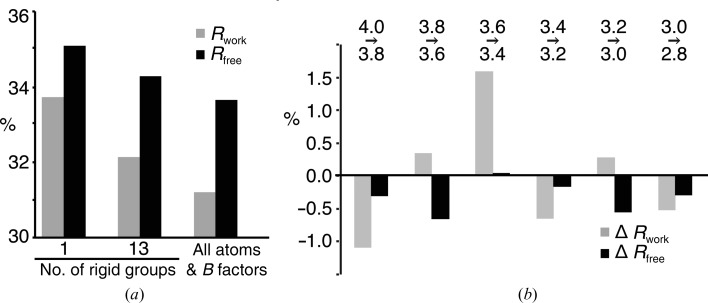
*R*_work_ and *R*_free_ values resulting from different refinement strategies. (*a*) *R* values after refinement with increasing numbers of parameters, including data to 4.0 Å resolution. (*b*) Differences in *R* values after refinement (all atom coordinates, individual *B* factors) depending on the chosen high-resolution data cutoff. Models were refined in parallel to both the lower and the higher resolution cutoff and the *R* values were compared at the lower cutoff. The range of lower data quality between 3.6 and 3.4 Å is probably caused by high background scattering owing to the solvent.

Nevertheless, we obtained a clear solution by MR using the synchrotron structure (PDB entry 3n8g; Bublitz *et al.*, 2013 ▸) with all ligands removed as a search model (see §[Sec sec1]1 for details; Fig. 1 ▸ *b*). An initial rigid-body refinement with increasing numbers of rigid groups was carried out in *PHENIX* using data to 4 Å resolution, and finally an all-atom and individual *B*-factor refinement yielding a decrease in the model *R* factors (Fig. 2 ▸ *a*). In order to determine the effective resolution limit of the data, we then carried out successive refinement runs with a stepwise inclusion of higher resolution shells, as suggested in recent work by Karplus & Diederichs (2012 ▸), Steuber *et al.* (2014 ▸) and Hattne *et al.* (2014 ▸) (Fig. 2 ▸ *b*). This analysis revealed an improvement of model *R* factors upon the inclusion of higher resolution data, justifying an extension of the data set to 2.8 Å resolution. Only the resolution range between 3.6 and 3.4 Å led to increased *R* factors, most likely owing to high solvent background scattering in this resolution range. The strategy of expanded resolution led to a final refined model with *R*_work_ and *R*_free_ values of 30.6 and 34.4%, respectively, at 2.8 Å resolution.

**Figure 3:**
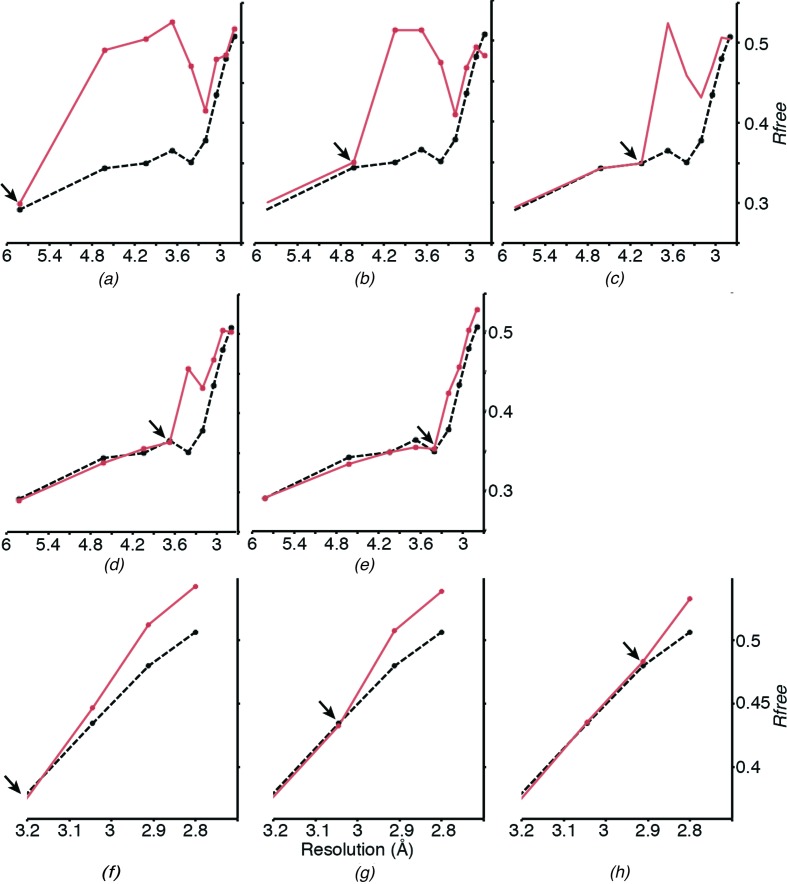
Plot of free *R* factor against resolution after refinement (all atom coordinates, individual *B* factors) to 2.8 Å resolution against the original data set (dashed black lines) and hybrid data sets (red lines) with scrambled intensities. The arrows indicate the beginning of the scrambled data at (*a*) 5.82 Å, (*b*) 4.62 Å, (*c*) 4.04 Å, (*d*) 3.67 Å, (*e*) 3.41 Å, (*f*) 3.21 Å, (*g*) 3.04 Å and (*h*) 2.91 Å. These cutoffs were chosen in accordance with the resolution shells used by *phenix.refine* to calculate the *R*_free_ values of the model.

As a control for the significance of the information in the low-multiplicity 2.8 Å resolution data, we generated data sets with randomly scrambled reflection intensities ranging from a wide shell of scrambled intensities (5.8–2.8 Å) to a narrow shell (2.9–2.8 Å) at high resolution only. We compared the statistics of refinement against these data with those derived from refinement against the unperturbed data. As seen in Fig. 3 ▸, the introduction of the scrambled data was clearly detectable as a deviation point in *R*_free_ values, even if only the highest resolution shell between 2.9 and 2.8 Å had been scrambled. This shows that the SFX intensities contain consistent signal. As a further analysis of the quality of the higher resolution data, we performed refinement of scrambled models to challenge the benefit and phase bias of a pre-refined model. Here, however, we found that the model refinement could not converge towards an improved model. Thus, the higher resolution data only improve map features when high-quality prior phases are available.

### 3.4. SERCA–Ca_2_–AMPPCP: analysis of bound ligands   

**Figure 4:**
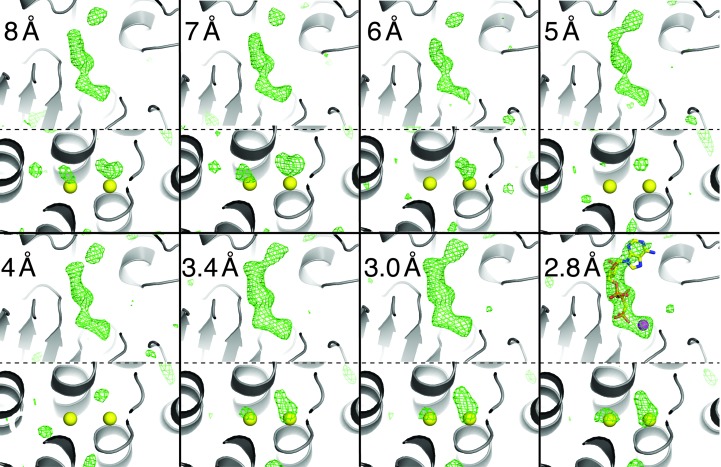
Electron density for SERCA ligands at different resolutions. Upper panels depict the nucleotide-binding site (region 1 in Fig. 1 ▸ *b*) and lower panels the Ca^2+^-binding site (region 2 in Fig. 1 ▸ *b*). Green mesh, *mF*_o_ − *DF*_c_ map after refinement without any ligands contoured at 3.0σ. Refinements with resolution cutoffs lower than 3.4 Å were restricted to 13 rigid-body groups, whereas refinements including data to 3.4 Å resolution or higher included individual atom coordinates and *B* factors. The expected ligand positions are superposed on all panels for Ca^2+^ and on the last panel for Ca^2+^–AMPPCP (PDB entry 3n8g). For a quantification of the density coverage around the ligands, see Supplementary Figs. S3 and S4.

After rigid-body refinement at low resolution, the *mF*_o_ − *DF*_c_ difference map of the refined ligand-free SERCA model already revealed a prominent peak at the nucleotide-binding site (site 1 in Fig. 1 ▸ *b* and Fig. 4 ▸) consistent with the presence of bound Ca^2+^–AMPPCP in the microcrystals and overlapping very accurately with the position of the Ca^2+^–AMPPCP ligand in the superposed synchrotron-derived model (Fig. 4 ▸, upper panels). As to be expected for valid data, the connectivity and the signal level of the electron density improved with increasing resolution, with all moieties of the ligand reaching a level of >3σ at 3.2 Å resolution and levelling off beyond 3 Å resolution (Supplementary Fig. S3). We were also able to detect the two bound Ca^2+^ ions located at the intramembranous binding sites I and II (site 2 in Fig. 1 ▸ *b*, lower panels of Fig. 3 ▸ and Supplementary Fig. S4). Importantly, the two calcium ions in the difference maps were only apparent when all atom coordinates and *B* factors were refined and data at 3.5 Å resolution or beyond were included in the refinement.

**Figure 5:**
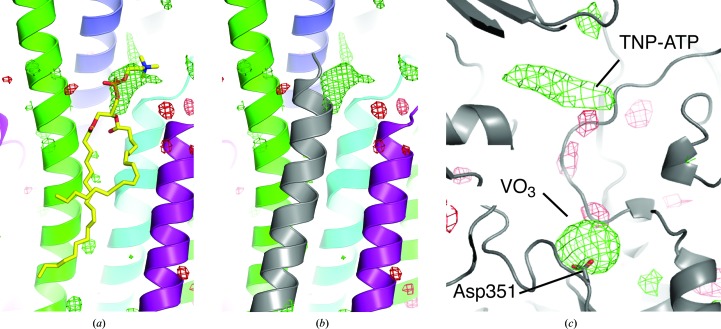
Electron density of SERCA ligands. A peak of ∼6σ in the *mF*_o_ − *DF*_c_ OMIT map after refinement without ligands (all atom coordinates, individual *B* factors) against data to 2.8 Å resolution overlaps with (*a*) a phosphatidylcholine modelled in the E1–ADP–AlF_4_ form (stick representation; PDB entry 2zbd; Toyoshima *et al.*, 2004 ▸) and (*b*) the N-terminal part of regulatory sarcolipin (grey cartoon; PDB entry 4h1w; Winther *et al.*, 2013 ▸). (*c*) *mF*_o_ − *DF*_c_ OMIT map (green, 3.0σ; red, −3.0σ) calculated to 5 Å resolution at the vanadate- and TNPATP-binding sites. The side chain of the catalytic residue Asp351 is shown in stick representation.

The *mF*_o_ − *DF*_c_ difference electron-density map furthermore revealed an additional ligand peak, which could be assigned to a bound phospholipid head group at the membrane interface between transmembrane helices M2, M6 and M9 (site 3 in Fig. 1 ▸ *b* and Figs. 5 ▸ *a* and 5 ▸ *b*). The position of the peak coincides with a lipid head group modelled into previous structures of SERCA in the Ca_2_E1 state (Toyoshima *et al.*, 2011 ▸) and the related Ca^2+^-occluded Ca_2_E1–ADP–AlF_4_^−^ complex (Picard *et al.*, 2007 ▸; Fig. 5 ▸ *a*). Interestingly, this groove was recently identified as the binding site for sarcolipin, a natural regulator of SERCA activity (Winther *et al.*, 2013 ▸; Toyoshima *et al.*, 2013 ▸; Fig. 5 ▸ *b*), pinpointing this region as a putatively generic modulatory site for lipids or regulatory proteins during the E1–Ca_2_E1P transition of the ATPase (Drachmann *et al.*, 2014 ▸).

The overall differences between the final room-temperature FEL-derived and the synchrotron-derived structure are small; the r.m.s.d. over all atoms is 0.46 Å. The average *B* factor of the FEL structure is slightly higher but quite comparable to that of the synchrotron structure (average *B* of 114 Å^2^ for 2.8 Å resolution refinement *versus* 97 Å^2^ for 2.6 Å resolution refinement).

### 3.5. SERCA–VO_3_–TNPATP   

Microcrystals of SERCA–VO_3_–TNPATP diffracted to 4 Å resolution (Supplementary Fig. S5*a*). We collected 1 371 609 images, 83 934 (6.1%) of which were classified as hits; 4910 of these (5.8%) could be indexed, notably revealing a crystal form with a different space group from that of macrocrystals grown under similar conditions (SFX, *P*42_1_2, *a* = *b* = 268, *c* = 114.5 Å, α = β = γ = 90°; synchrotron, *P*2_1_, *a* = 130, *b* = 95, *c* = 135 Å, β = 107°). MR was successfully carried out with an aluminium tetrafluoride-stabilized structure (PDB entry 3n5k) with the ligands omitted, which we anticipated to be in the same conformational state as the orthovanadate-bound structure. After comparing *R* values from rigid-body refinement at stepwise increasing resolution, we set the ‘effective’ high-resolution cutoff for this data set to 5 Å (see Supplementary Fig. S6).

After rigid-body refinement of the MR-derived initial model (ligand-free; see §[Sec sec2]2 for details) including data to 5 Å resolution, we calculated an *mF*_o_ − *DF*_c_ difference map. The two strongest positive peaks (∼12σ and ∼10σ) were observed at the phosphorylation sites of the two molecules in the asymmetric unit, *i.e.* coinciding with the anticipated site of vanadate binding. Furthermore, weaker peaks (∼4.9σ and ∼4.5σ) were apparent for the bound TNPATP nucleotide analogue in both molecules in the asymmetric unit. We also additionally found a weak positive peak (3.4σ) overlapping with the vanadate position in one of the two molecules in an anomalous difference Fourier map calculated to 5 Å resolution. However, neither this peak nor several other peaks in the anomalous difference map which could be assigned to overlapping positions of S atoms in the model clearly stood out from the noise level, indicating that a higher multiplicity or higher resolution is required for a robust anomalous signal for moderate anomalous scatterers to emerge from SFX data. Nevertheless, this example shows that it is possible to identify the signal of TNPATP and vanadate as ligands with SFX data extending to only 5 Å resolution with a multiplicity of ∼120. During the preparation of this manuscript, higher resolution synchrotron data were obtained for the SERCA–VO_3_–TNPATP complex, confirming the overall conformation of the enzyme and the binding sites of both vanadate and TNPATP in our low-resolution structure (Clausen *et al.*, manuscript in preparation).

### 3.6. SsZntA–AlF_4_   

We started our microcrystallization attempts based on the conditions for macroscopic Zn^2+^-ATPase crystals with an elongated hexagonal plate-like morphology: approximately 100 µm in the largest dimension but only a few micrometres in thickness. The microcrystals of SsZntA–AlF_4_ were approximately 5 µm long rods that diffracted to ∼4 Å resolution (Supplementary Fig. S5*b*). We collected ∼280 000 images, 16 358 of which were classified as hits, but only 55 of these (0.3%) could be indexed. Interestingly, the microcrystals again displayed a new space-group symmetry (SFX, *P*422, *a* = *b* = 58, *c* = 320 Å, α = β = γ = 90°; synchrotron, *C*222_1_, *a* = 77, *b* = 83, *c* = 320 Å, α = β = γ = 90°). The long *c* axis of 320 Å however probably caused the very low indexing rate owing to closely adjacent reflections on the diffraction images. By performing a six-dimensional search to determine the optimal values for *a*, *b*, *c*, α, β and γ, we attempted to determine a range of values which led to the highest indexing rates, but with a maximum of only 55 indexed images the data set did not support successful phasing by molecular replacement. Recently, higher resolution synchrotron data were obtained from macrocrystals of the Zn^2+^-ATPase and the resulting crystal structures are discussed elsewhere (Wang *et al.*, 2014 ▸).

## 4. Discussion   

SFX experiments exploiting the femtosecond pulse duration and outstanding peak brilliance of XFELs hold great promise for challenging crystal structure determinations. Here, we demonstrate the feasibility of this method for ligand screening, testing different ligand complexes of microcrystalline P-type ATPases from native and recombinant sources.

The resolution of the collected SFX data for SERCA–Ca_2_–AMPPCP was slightly lower than that of synchrotron data from macrocrystals (Sørensen *et al.*, 2004 ▸), likely owing to a small time window for optimal microcrystal quality. Only a 24 h difference in the age of the crystal sample led to weaker overall diffraction and a higher number of overlapping diffraction patterns, indicating crystal clustering. Optimization of the crystallization conditions is therefore likely to allow a further improvement of the observed diffraction and should be a general focus when growing microcrystals. Furthermore, the fact that in all three cases the microcrystals grew much faster than corresponding macrocrystals accommodates a ligand-screening scenario well. In other cases we did not succeed, such as for Na^+^,K^+^-ATPase crystals, for which synchrotron radiation provided a 4.3 Å resolution data set (Nyblom *et al.*, 2013 ▸). For those crystals we observed only 20 Å resolution at best from microcrystals (data not shown).

Nevertheless, valid structural information can be extracted from SFX data even at resolution ranges where 〈*I*/σ(*I*)〉 is below 1 or CC_1/2_ approaches zero (Fig. 5 ▸) if good phases are available. *CrystFEL* applies a global resolution cutoff to all frames. If the data set contains diffraction patterns of weakly or poorly diffracting crystals, this can result in the inclusion of very weak high-resolution intensities. This not only contributes to the low 〈*I*/σ(*I*)〉 values mentioned above, but may also affect the intensity distribution and Wilson plot (see Sawaya *et al.*, 2014 ▸). *CrystFEL* uses Monte Carlo methods to obtain merged intensities. Their accuracy strongly depends on the number of observations to average out the various fluctuations in the individual measurements. Indeed, very high multiplicities were required for *de novo* phasing, which requires highly accurate structure-factor measurements (Barends *et al.*, 2014 ▸). However, the accuracy of the measured intensities does not have to be very high to observe features in a phased Fourier map (possibly even an anomalous map). Almost counterintuitively, Fourier maps can show details that one would not expect to be able to see given the accuracy of the data (Henderson & Moffat, 1971 ▸). This is the reason why the high-resolution data of the SERCA–Ca_2_–AMPPCP complex provide useful information despite their poor 〈*I*/σ(*I*)〉 value and their low multiplicity of measurements [in our case 〈*I*/σ(*I*)〉 and multiplicity in the highest resolution shell are as low as 0.39 and 4.2, respectively; Table 1 ▸].

**Figure 6:**
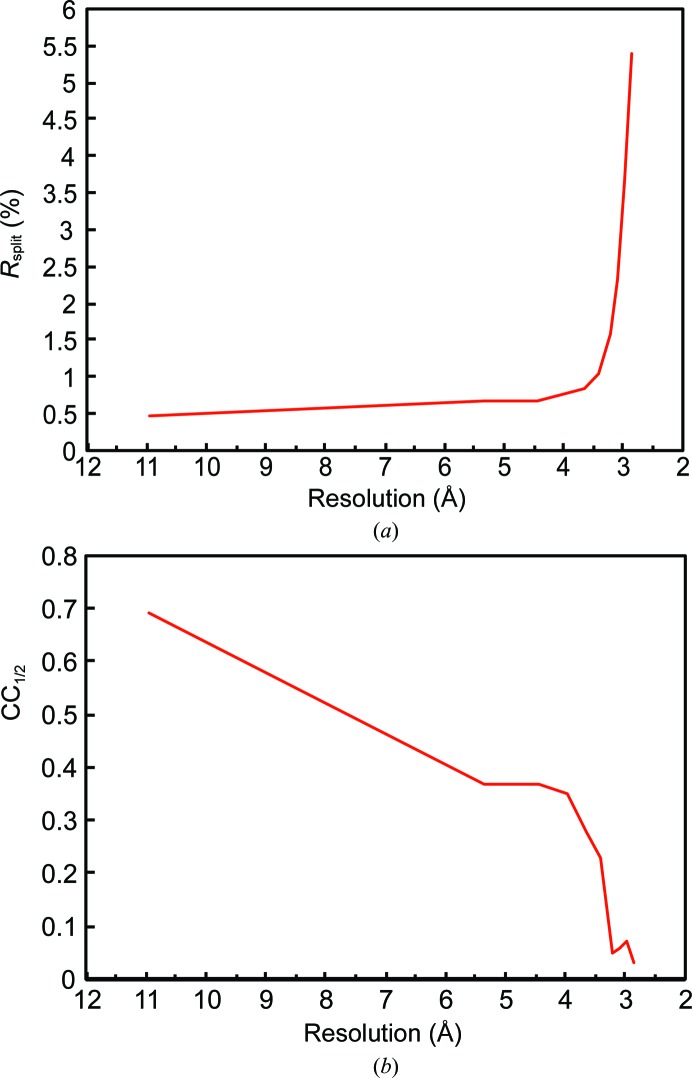
Statistics for SFX data from SERCA–Ca_2_–AMPPCP. (*a*) *R*_split_ and (*b*) correlation coefficient between random half data sets, CC_1/2_, against resolution.

Importantly, we also demonstrate that the inclusion of weak observations contributes favourably to model refinement compared with simulated random data (Fig. 3 ▸), similar to the findings of a recent analysis by Evans & Murshudov (2013 ▸). We therefore suggest that once the initial phases have been obtained, the ‘effective resolution’ should be determined empirically for each data set by the stepwise inclusion of higher resolution shells, comparing *R* values after a standardized refinement protocol. The number of refined parameters can then be adapted to the available amount of data for the determined resolution range. If known ligands are available, the analysis of unbiased difference electron-density maps of stepwise-increased resolution cutoffs can give valuable information about the ‘effective resolution’. In our test case of SERCA–Ca_2_–AMPPCP, we observe a levelling off of the density signal between 3.0 and 2.8 Å resolution, meaning that from some point within this range there is no additional signal available. However, since we had a clear indication of few but strong diffraction spots at 2.8 Å resolution (see Fig. 1*a*), we decided to include data to 2.8 Å resolution even though the high-resolution shell has poor crystallographic precision indicators such as an 〈*I*/σ(*I*)〉 of only 0.39, an *R*_split_ of >500% and a CC_1/2_ heading towards zero. Nevertheless, 18.3% of the data do still have an 〈*I*/σ(*I*)〉 value above 1 in the highest resolution shell. *R*_split_ and CC_1/2_ have been suggested as useful indicators of significant information content of crystallo­graphic data (Karplus & Diederichs, 2012 ▸), and other SFX studies report values of between 53% and 162% for *R*_split_ and 0.27 and 0.37 for CC_1/2_ in the highest resolution shells (Sawaya *et al.*, 2014 ▸; Johansson *et al.*, 2013 ▸; Liu *et al.*, 2013 ▸). In our case, the CC_1/2_ value reaches a minimum at ∼3.0 Å (Fig. 6 ▸). When calculating the actual significance of CC_1/2_ values with respect to the number of reflections in the highest resolution shell for data truncated at 3.0, 2.9 and 2.8 Å resolution, we find that the Student’s t-test *P*-values oscillate between significance at the 1% level and the 7% level (*P* = 0.0675 at 3.0 Å, *P* < 0.00001 at 2.9 Å and *P* = 0.0698 at 2.8 Å). Johannsson *et al.* (2014 ▸) report a smooth decline of CC_1/2_ to zero, making it similarly difficult to determine a definite limit of significance. They finally used a decrease in the quality of electron density as an indicator to truncate the data at a CC_1/2_ of 0.3. Since in our case the ligand electron-density levels do not decline (Fig. 4 ▸ and Supplementary Figs. S3 and S4) even at a CC_1/2_ of 0.08 in the 2.9–2.8 Å shell, we decided to include all data showing any weak indication of significance. In addition, both the *R*_free_ values (Fig. 2 ▸ *b*) and the scrambled data comparisons (Fig. 3 ▸) confirm that the inclusion of these weak data did introduce detectably more signal than noise. Specifically, if we had not included data to 2.8 Å resolution it would not have been possible to reliably detect the two bound Ca^2+^ ions in the structure. If good phases are available, the information content of difference Fourier maps can be much higher than immediately expected from the data quality. This is typically the case for, *e.g.*, ligand-soaked crystals where a higher resolution template structure is available, or when averaging by non­crystallographic symmetry can be applied.

Another demonstration of the information available from SFX data even at low resolution is the fact that we observed a number of peaks in a phased anomalous difference map calculated with the 5 Å resolution SERCA–VO_3_–TNPATP data that could be assigned to anomalous scatterers. Even though the map was also very noisy, we assume that a higher redundancy would have been sufficient to provide a robust anomalous signal. At the photon energy used (6.0 keV) the tabulated *f*′/*f*′′ of vanadium is ∼1.3/∼3.5 e^−^, and the expected signal makes a 0.5–0.9% contribution to the structure-factor amplitudes. This contribution is similar to the situation reported recently for photosystem II (PSII; Kern *et al.*, 2014 ▸; at 7 keV and 4.9 Å resolution the expected signal makes a 0.7–1.0% contribution to the structure-factor amplitudes), which however has four manganese ions and one calcium ion in the oxygen-evolving complex, making this effectively a relatively easily detectable superatom at low resolution. The overall multiplicity of observations was ∼100 in the case of PSII (3F data) and ∼120 in the case of SERC–VO_3_–TNPATP. This finding will encourage screening projects in which anomalous scatterers can be used to identify and analyse ligand-binding sites, such as in drug discovery.

We expect that ongoing developments in data-analysis programs such as profile fitting and post-refinement will not only significantly improve data quality but also reduce the number of required diffraction patterns and thus the sample and XFEL beamtime. Indeed, a recent comparison between data analysis with *CrystFEL* and *nXDS* (Kabsch, 2014 ▸), which uses two-dimensional profile fitting for estimating the observed reflection intensity and subsequent post-refinement of the correction factors relating each observation to its unique squared structure-factor amplitude, shows that comparable data can be obtained using *nXDS* from far fewer images (Botha *et al.*, 2015 ▸). Moreover, recent developments in sample-delivery methods such as the use of high-viscosity extrusion injectors (Weierstall, 2014 ▸; Botha *et al.*, 2015 ▸) can greatly reduce sample consumption owing to low flow rates. They are particularly well suited when the proteins investigated crystallize in the lipidic cubic phase (LCP) or are compatible with the recently described grease matrix (Sugahara *et al.*, 2015 ▸).

An additional benefit of liquid jet-based sample delivery is that the crystals are kept at ambient temperature during SFX measurements, allowing analysis of the structural distributions and dynamics under more native conditions. As recently demonstrated (Fraser *et al.*, 2011 ▸), this is important since structural dynamics and consequently the observed conformations are often temperature-dependent. Thus, monitoring ambient temperature conformational ensembles by X-ray crystallography can reveal motions crucial for catalysis, ligand binding and allosteric regulation (Fraser *et al.*, 2011 ▸). This can be particularly important for proteins that display a wide range of functionally important conformations such as G-protein coupled receptors or the P-type ATPases described here.

In conclusion, our data show that SFX is a valuable approach for efficient ligand screening with membrane proteins since a low multiplicity of measurements is sufficient to identify small features in phased difference electron-density maps. Our results show that the effective resolution limit of such data needs to be determined with great care. We strongly encourage the inclusion of weak high-resolution data reaching the limit of statistical significance and to empirically test their influence on the quality of the resulting molecular model to ensure that no valid data are discarded.

## Supplementary Material

PDB reference: P-type ATPase–ligand complex, 4xou

Supplementary Figures S1-S6 and Supplementary Tables S1-S6. DOI: 10.1107/S2052252515008969/jt5009sup1.pdf

## Figures and Tables

**Table 1:** Data-collection and refinement statistics Values in parentheses are for the outer shell except where indicated otherwise.

	SERCACa_2_AMPPCP	SERCAVO_3_TNPATP	SsZntAAlF_4_
Data-collection and processing			
X-ray energy (keV)	6	6	6
Collected frames	761730	1371609	280281
Diffraction hits†	23016 (3.0)	83934 (6.1)	16358 (5.8)
Indexed frames‡	4069 (17.8)	4910 (5.8)	55 (0.3)
Space group	*C*2	*P*42_1_2	*P*422
*a*, *b*, *c* ()	162, 76.3, 151	268, 268, 114.5	58, 58, 320
, , ()	90, 109.0, 90	90, 90, 90	90, 90, 90
Highest resolution observed ()	2.8	4	4
Resolution range for processing ()	59.92.8 (2.92.8)	57.255.00 (5.185.00)	n.a.§
Unique reflections	42416 (3652)	18601¶ (1813)	n.a.§
Multiplicity	17.3 (4.2)	124 (117)	n.a.§
*I*/(*I*)	1.13 (0.39)	2.03 (0.34)	n.a.§
*R*_split_ †† (%)	62.3 (538)	22.5 (424)	n.a.§
CC_1/2_	0.70 (0.03)	0.97 (0.16)	n.a.§
CC*	0.90 (0.25)	0.99 (0.53)	
Anomalous CC‡‡	n.a.	0.06 (0.03)	n.a.§
Completeness (%)	98.1 (85.5)	99.91 (100)	n.a.§
Molecular replacement			
Search-model PDB code§§	3n8g	3n5k	
Rotation-function *Z*-score (ensemble 1/2)	8.4	2.3/1.8	
Translation-function *Z*-score (ensemble 1/2)	10.6	11.5/20.4	
Log-likelihood gain	4295	991	
Refinement			
Resolution ()	602.80 (2.912.80)		
No. of reflections	41471 (3500)		
Final *R*_cryst_/*R*_free_ (%)	30.4/34.3 (47.5/49.9)		
No. of non-H atoms			
Protein	7671		
Ca^2+^	3		
K^+^	1		
AMPPCP	31		
Total	7706		
R.m.s. deviations			
Bonds ()	0.006		
Angles ()	0.762		
Average *B* factors (^2^)			
N domain	97.7		
P domain	97.6		
A domain	114		
TM domain	130.3		
Ca^2+^	113		
K^+^	91.0		
AMPPCP	88.6		
Ramachandran plot			
Most favoured (%)	95.1		
Allowed (%)	4.4		

† Numbers in parentheses are the percentage of the total collected frames.

‡ Numbers in parentheses are the percentage of indexed frames.

§ The Zn^2+^-ATPase data set was not merged because the number of indexed patterns was too low to obtain meaningful merged data.

¶ The according numbers of unique reflections when merged with Friedel pairs treated as separate reflections is 31576 (3262).

†† *R*_split_ is defined in *CrystFEL* as the *R* factor between data sets calculated from two randomly chosen halves of the data, corrected for the decrease in multiplicity caused by dividing the data into halves: *R*_split_ = 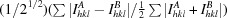.

‡‡ Determined from processing the data set with separated Friedel mates.

§§ See [Sec sec2]2 for details of how the search models were prepared to remove model bias.
