# Supplementary material for: Structural studies of P-type ATPase–ligand complexes using an X-ray free-electron laser
Source: IUCrJ. 2015 Jun 11;2(Pt 4):409–20. doi: 10.1107/S2052252515008969 (PMC4491313; doi:10.1107/S2052252515008969)
Supplement: Supplementary file 1 [file m-02-00409-sup1.pdf]

IUCrJ

Volume 2 (2015)

Supporting information for article:

Structural studies of P-type ATPase ligand complexes using an X-ray free-electron laser

Maike Bublitz, Karol Nass, Nikolaj D. Drachmann, Anders J. Markvardsen, Matthias J. Gutmann, Thomas R. M. Barends, Daniel Mattle, Robert L. Shoeman, R. Bruce Doak, Sébastien Boutet, Marc Messerschmidt, Marvin M. Seibert, Garth J. Williams, Lutz Foucar, Linda Reinhard, Oleg Sitsel, Jonas L. Gregersen, Johannes D. Clausen, Thomas Boesen, Kamil Gotfryd, Kai-Tuo Wang, Claus Olesen, Jesper V. Møller, Poul Nissen and Ilme Schlichting

Figure S1 Statistics for SFX data from SERCA-Ca_2-AMPPCP: CC* against resolution.

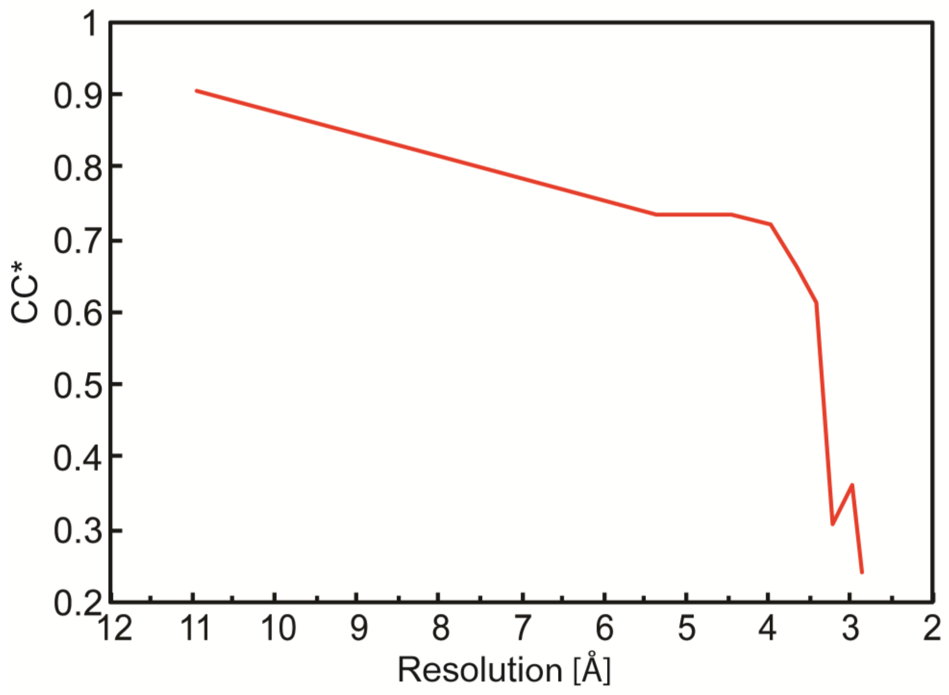

Figure S2 Data-to-parameter ratio (ratio between the number of unique reflections and the number of refined parameters) for different refinement strategies, depending on the resolution cutoff applied.

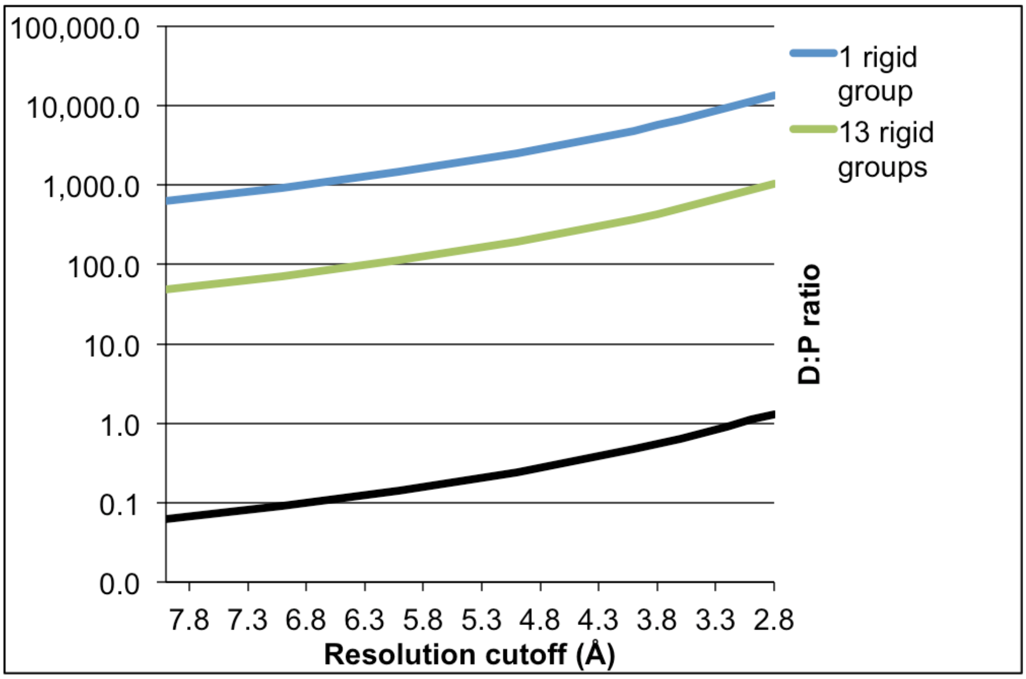

Figure S3 Coverage of the AMPPCP ligand in the SERCA-Ca_2-AMPPCP complex by positive difference density. *m*Fo-*D*Fc difference map values of different moieties of the AMPPCP ligand, dependent on resolution cutoff. At ~3.2 Å, all moieties of the ligand reach values above 3 σ, and values increase up to 3.0 or 2.8 Å resolution. Values were determined with PHENIX (phenix.map_value_at_point), yielding the eight-point interpolated density value of the *m*Fo-*D*Fc map computed at atomic center of the superposed (from 3N8G) coordinates of the α-, β-, and γ-phosphate atom, the associated Ca^{2+} ion and the respective center of mass of the adenine and the ribose moiety, as determined with PDBSET.

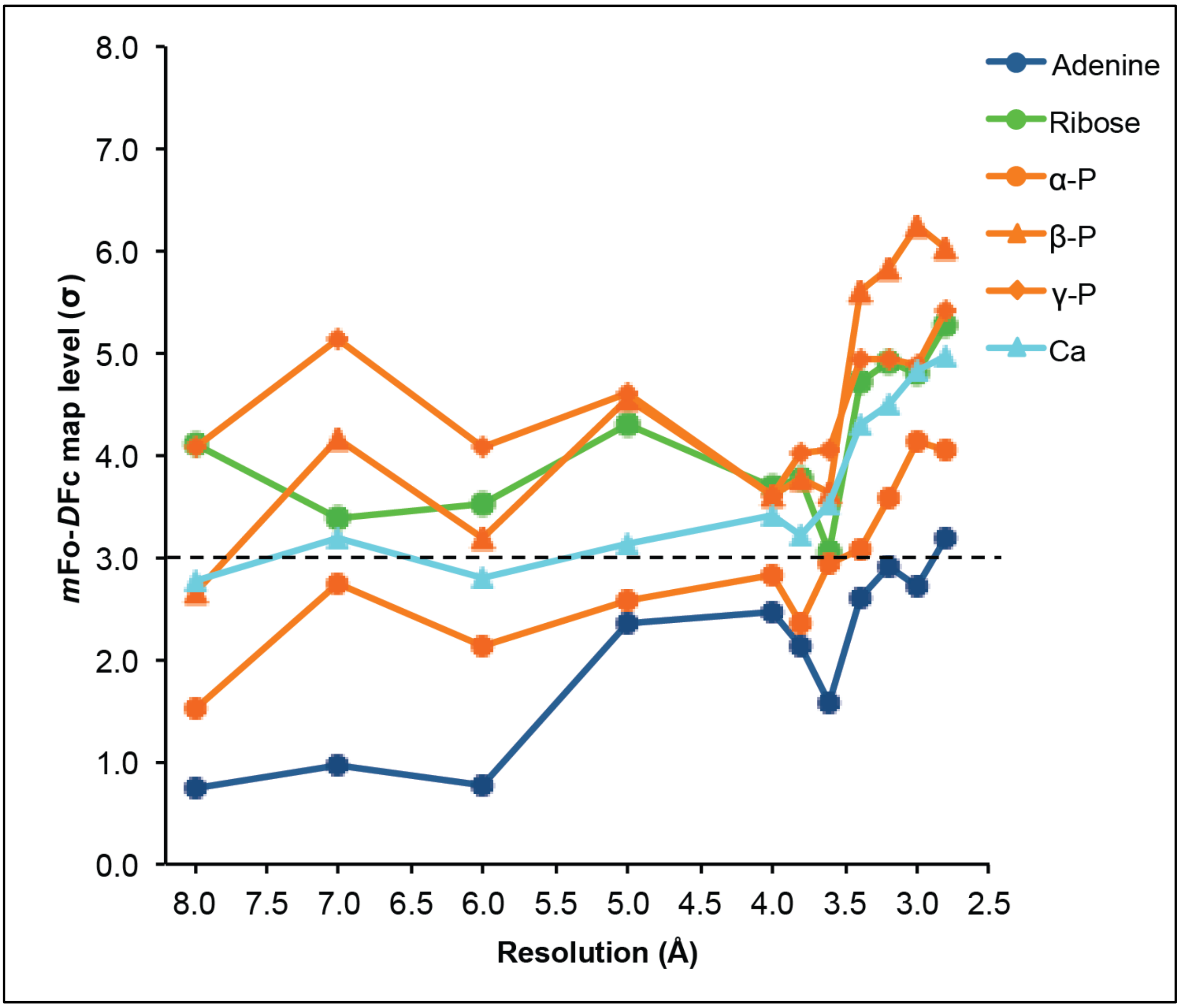

Figure S4 Positive difference density at the two bound Ca^{2+} ions. *m*Fo-*D*Fc difference map level of the two bound Ca^{2+} ions in the SERCA-Ca_2-AMPPCP complex, dependent on resolution cutoff. The signal increases sharply after switching to an all-atom refinement strategy at 3.4 Å data cutoff. Only with data extending to 2.8 Å, both Ca^{2+} ions reach a level above 2.8 σ. Levels were determined with PHENIX (phenix.map_level_at_point), using the coordinates of the Ca^{2+} ions superposed from PDB ID 3N8G.

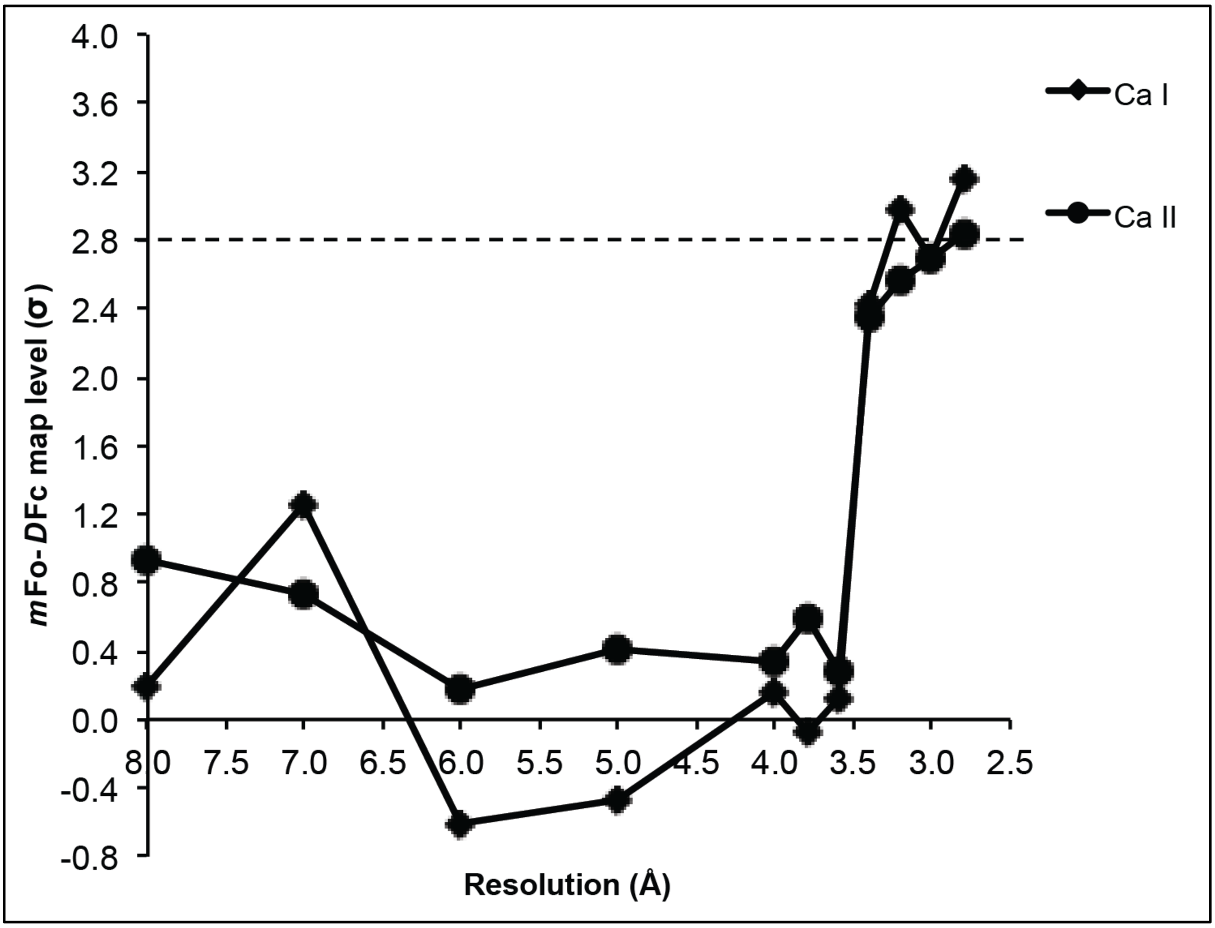

Figure S5 SFX diffraction patterns. **(A)** SERCA-VO_3-TNPATP **, (B)** SsZntA-AlF_4

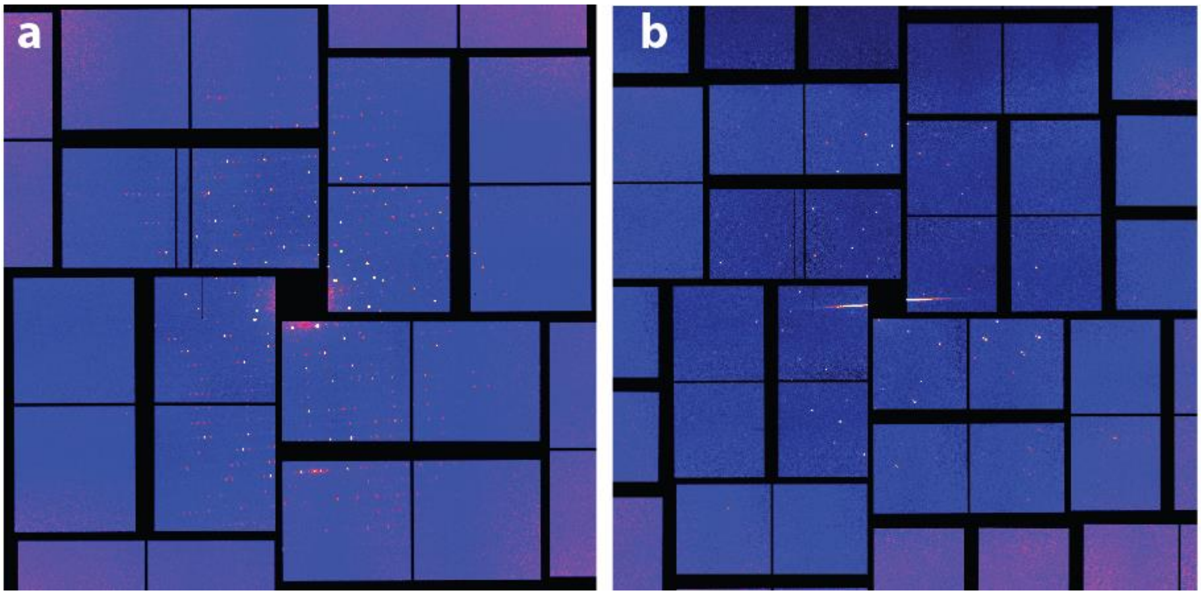

Figure S6 R*free* values from different rigid body refinement strategies of SERCA-VO_3-TNPATP at increasing resolution cutoffs. Values start to increase sharply beyond a cutoff at 5 Å, which was accordingly chosen as the effective resolution of the dataset.

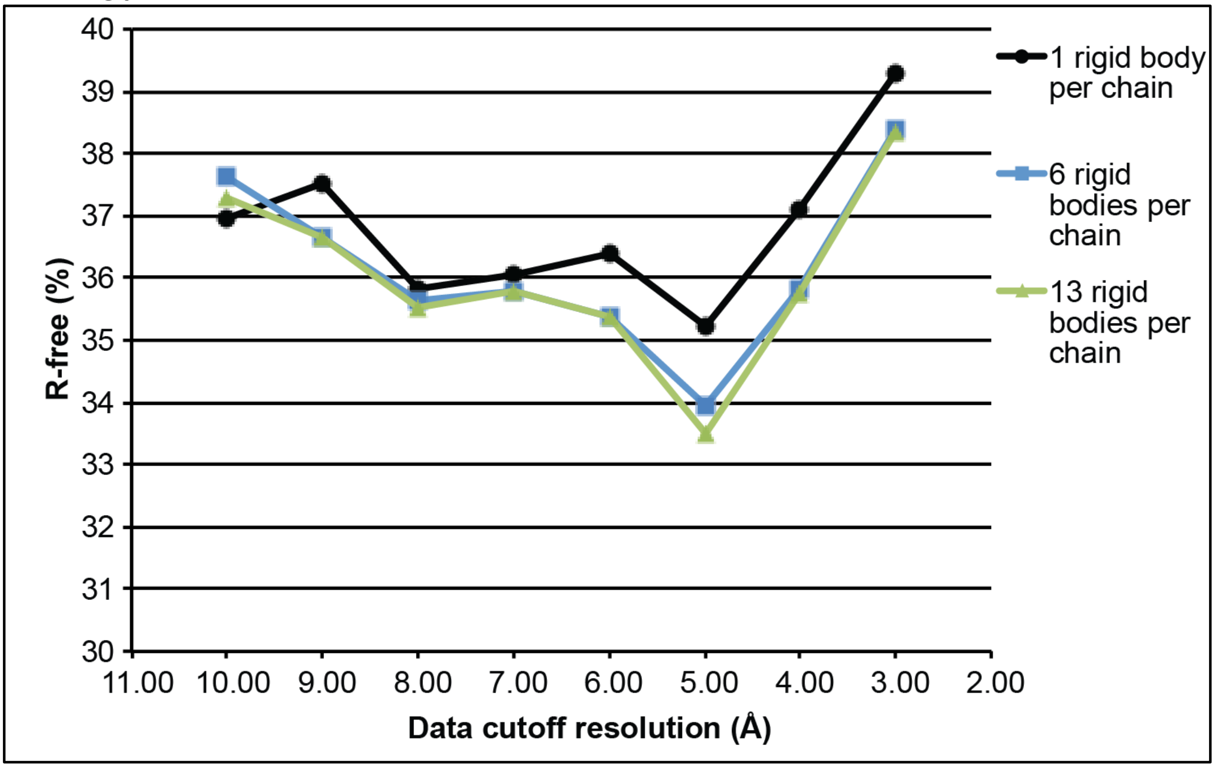

Table S1 Macro- and microcrystal growth conditions and unit cell parameters[a]

	Macrocrystals			Microcrystals		
	Method	Precipitant solution	Space group; Unit cell parameters	Batch size	Precipitant solution	Space group; Unit cell parameters
**SERCA-Ca$_2$-AMPPCP**	Hanging drop[1]	8 % PEG6000<br>200 mM NaOAc<br>15 % glycerol<br>4 % *tert*-butanol<br>5 mM *β*-ME	*C*2<br>*a*=162 Å<br>*b*= 76 Å<br>*c*=151 Å<br>*β*=108°	30 µL	21.5 % PEG6000<br>200 mM NaOAc<br>15 % glycerol<br>4 % *tert*-butanol<br>5 mM *β*-ME	*C*2<br>*a*=162 Å<br>*b*=76 Å<br>*c*=151 Å<br>*β*=109°
**SERCA-VO$_3$-TNPATP**	Hanging drop	20-23 % PEG2000 MME<br>10 % glycerol<br>100 mM NaCl<br>3 % *tert*-butanol	$P2_1$<br>*a*=130 Å<br>*b*=95Å<br>*c*=136 Å<br>*β*=107°	40 µL	28 % PEG2000 MME<br>10 % glycerol<br>100 mM NaCl<br>3 % *tert*-butanol	$P42_12$<br>*a*=*b*=268 Å<br>*c*=115 Å
SsZntA-AlF$_4$	Hanging drop[2]	300 mM LiOAc<br>16 % PEG2000 MME<br>10 % glycerol<br>6 % MPD<br>5 % D-sorbitol<br>5 mM β-ME	$C222_1$<br>*a*=77 Å<br>*b*=83 Å<br>*c*=320 Å	100 µL	500 mM LiOAc<br>15 % PEG2000 MME<br>10 % glycerol<br>3 % *tert*-butanol<br>5 % D-sorbitol<br>5 mM *β*-Me	*P*422<br>*a*=*b*=58 Å<br>*c*=320 Å

[a]Abbreviations: SERCA, sarco(endo)plasmic reticulum calcium ATPase; AMPPCP, 5’adenylyl (β,γ-methylene)diphosphonate; TNPATP, 2',3'-*O*-(2,4,6-Trinitrophenyl)adenosine-5'-triphosphate; VO3, orthovanadate; SsZntA, *Shigella sonnei* Zn^{2+}-ATPase; AlF_4, aluminium tetrafluoride; PEG, polyethylene glycol; MME, monomethyl ether.

[1] Sørensen, T. L.-M., Møller, J. V. & Nissen, P. (2004). *Science*, **304**, 1672–1675.

[2] Wang, K., Sitsel, O., Meloni, G., Autzen, H. E., Andersson, M., Klymchuk, T., Nielsen, A. M., Rees, D. C., Nissen, P. & Gourdon, P. (2014). *Nature*, **514**, 518-522.

Table S2 SERCA-Ca_2-AMPPCP data statistics in resolution bins

# Reflections	Possible	Compl. (%)	# Measurements	Multiplicity	SNR	Resolution shell center (Å)
4463	4463	100.00	131520	29.5	2.70	10.96
4348	4348	100.00	105684	24.3	1.94	5.34
4359	4359	100.00	99703	22.9	1.78	4.46
4299	4299	100.00	96618	22.5	1.47	3.98
4327	4327	100.00	105489	24.4	1.28	3.66
4276	4276	100.00	80029	18.7	0.88	3.42
4328	4333	99.88	52826	12.2	0.45	3.23
4284	4310	99.40	36652	8.6	0.27	3.08
4080	4252	95.95	23172	5.7	0.15	2.96
3652	4272	85.49	15336	4.2	0.39	2.85

Table S3 SERCA-Ca_2-AMPPCP Rsplit and CC ½ in resolution bins

Rsplit (%)	CC 1/2	# Reflections	Resolution shell center (Å)
45.84	0.69	4460	10.93
66.87	0.37	4343	5.34
68.96	0.37	4344	4.46
77.65	0.35	4289	3.98
84.92	0.28	4325	3.66
105.56	0.23	4251	3.42
159.35	0.05	4091	3.23
231.98	0.06	3573	3.08
368.35	0.07	2396	2.96
538.29	0.03	1316	2.85

Table S4 SERCA-VO_3-TNPATP data statistics in resolution bins

# Reflections	Possible	Compl. (%)	# Measurements	Multiplicity	SNR	Resolution shell center (Å)
2017	2022	99.75	348023	172.5	6.57	18.12
1889	1900	99.42	216544	114.6	4.21	9.52
1871	1871	100.00	253057	135.3	3.31	7.97
1850	1850	100.00	239383	129.4	2.19	7.11
1850	1850	100.00	216591	117.1	1.35	6.53
1846	1846	100.00	234260	126.9	1.01	6.11
1827	1827	100.00	205324	112.4	0.64	5.78
1825	1825	100.00	181473	99.4	0.39	5.51
1813	1813	100.00	206701	114.0	0.32	5.28
1813	1813	100.00	211134	116.5	0.34	5.09

Table S5 SERCA-VO_3-TNPATP Rsplit and CC ½ in resolution bins

Rsplit/%	# Reflections	CC	Resolution shell center (Å)
14.11	2013	0.97	18.10
25.03	1848	0.89	9.52
28.90	1871	0.89	7.97
42.33	1850	0.85	7.11
69.28	1852	0.77	6.53
103.33	1844	0.56	6.11
188.69	1814	0.34	5.78
326.31	1811	0.30	5.51
394.95	1813	0.22	5.28
424.01	1813	0.16	5.09

Table S6 SERCA-VO_3-TNPATP anomalous data statistics

**CCano[3,a]**	**# Reflections**	**Resolution shell center (Å)**
0.03	2890	17.79
0.15	2994	9.52
0.07	3154	7.97
0.00	3192	7.11
0.00	3192	6.53
0.01	3238	6.11
0.01	3208	5.77
0.00	3210	5.51
0.02	3236	5.28
0.04	3262	5.09

[a] CCano was determind with CrystFEL using the following formula:

CCano = $\frac{1/(n-1)\sum(x_i-\hat{x})(y_i-\hat{y})}{\sqrt{1/(n-1)\sum(x_i-\hat{x})^2}\sqrt{1/(n-1)\sum(y_i-\hat{y})^2}}$, with x and y representing the anomalous differences $\Delta F = (F^+) - (F^-)$ of two half-sets of the data.

[3] White, T. A., Kirian, R. A., Martin, A. V., Aquila, A., Nass, K., Barty, A. & Chapman, H. N. (2012). *J. Appl. Crystallogr*. **45**, 335–341.
